# Image-space compensation of inter-crystal scattering in PET using a neural network based filter

**DOI:** 10.1186/s40658-026-00837-9

**Published:** 2026-01-25

**Authors:** Dóra Varnyú, Ákos Rábely, László Szirmay-Kalos

**Affiliations:** 1 Mediso Medical Imaging Systems, Laborc u. 3., Budapest, 1037 Hungary; 2 Department of Control Engineering and Information Technology, Budapest University of Technology and Economics, Műegyetem rkp. 3., Budapest, 1111 Hungary

**Keywords:** Positron emission tomography, Inter-crystal scattering, Image filtering, Neural network, OS-EM, Skew normal distribution

## Abstract

****Background**:**

In positron emission tomography (PET), gamma photons arriving at the detector ring may undergo one or more Compton scattering events, potentially reaching a different scintillation crystal than its initial interaction point. This phenomenon, known as inter-crystal scattering (ICS), can lead to incorrect line of response (LOR) assignments, introducing spatial blurring and noise in the reconstructed image. Existing ICS correction methods include energy-based heuristics, Compton kinematics, Monte Carlo simulation, and deep learning to recover the first interaction position of the photons in the detector. These corrections are performed in the LOR domain, since that is where ICS manifests. However, in ordered-subset expectation-maximization (OS-EM) reconstruction, only a subset of LORs is processed at each iteration, making LOR-domain corrections difficult to apply on-the-fly.

****Methods**:**

We propose a novel ICS correction technique performed entirely in the image domain. A neural network is trained to predict spatially-varying 3D filter kernels that model the blurring effect of ICS across the image volume. These kernels are applied to the PET image estimate prior to each OS-EM forward projection. We evaluated our method on simulated and real PET data using two network variants: one predicting full 3D kernels (ICS-Net-direct), and another using a compact skew normal representation (ICS-Net-skewnorm).

****Results**:**

Both ICS-Net-direct and ICS-Net-skewnorm significantly improved the spatial resolution and the contrast of the output image, while also being computationally efficient. We found that ICS-Net-skewnorm is better suited for structural, symmetric reconstruction objects, while ICS-Net-direct performs best in complex, real-world scenarios.

****Conclusions**:**

The proposed image-domain ICS correction technique enables efficient and effective compensation of inter-crystal scattering for OS-EM reconstruction. Network selection should be guided by the target imaging scenario to maximize performance.

## Background

Positron emission tomography (PET) is a widely used nuclear imaging technique for visualizing metabolic activity in the body [1, 2]. It begins with the administration of a radiopharmaceutical, which accumulates in target tissues and undergoes positron decay, producing pairs of gamma photons that travel in nearly opposite directions. If the photons collide into the ring of scintillator crystals around the patient at the same time, the system records a *coincidence event*, and a *line of response* (LOR) is formed between the two activated crystals. PET image reconstruction aims to estimate the 3D distribution of the radiopharmaceutical by analyzing the statistical patterns of these LORs.

**Fig. 1:**
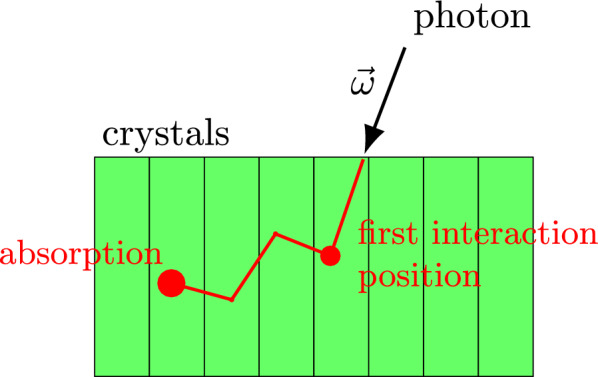
A photon may undergo one or more scattering events in the detectors before being absorbed, potentially reaching a different crystal than it initially interacted with

However, photon interactions within the detectors are not always straightforward. Before being absorbed, a photon may undergo one or more scattering events, potentially reaching a different detector crystal than its initial interaction point (Fig. 1). This mislocalization can lead to incorrect LOR assignments, introducing spatial blurring and noise in the reconstructed image.

Several strategies have been proposed to correct for inter-crystal scattering (ICS), mainly focusing on locating the first interaction position of the photon and the detector ring so that the true LOR can be identified [3].

The simplest solution measures the energy absorbed by each crystal. Comanor et al. [4] compared three methods for recovering the first interaction position during ICS: selecting the crystal with the highest energy deposition, selecting the one with the second-highest energy, and a joint strategy based on the energy difference between the crystals. This joint method selected the maximum energy crystal if the energy difference was higher than a predefined threshold; otherwise, it selected the second-highest. Their results showed that choosing the maximum energy crystal alone resulted in a 22% misidentification rate, whereas both the second-highest energy method and the joint method reduced the error to 12%.

Rafecas et al. [5] proposed several ICS correction algorithms aimed at estimating the correct photon path using Compton kinematics and the Klein-Nishina formula. Their best solution was a modified Compton kinematics scheme that applied maximum energy selection under certain circumstances. This hybrid method identified the first interaction position correctly with 59.3% probability, while the purely maximum energy selection method achieved only 50.6%.

Another approach is to use statistical optimization algorithms, such as Bayesian or maximum likelihood (ML) methods to estimate the first interaction position. These strategies can achieve better accuracy via incorporating prior knowledge on Compton kinematics. Gross-Weege et al. [6] proposed a ML solution based on comparing the expected and measured light distributions, using probability density functions (PDFs) pre-calculated from single photon interaction models. This achieved 19% better sensitivity than using simple averaged interaction position information.

In our previous work [7], we incorporated ICS into the system matrix of the maximum-likelihood expectation-maximization (ML-EM) reconstruction algorithm. The main idea was to decompose the system matrix into the product of simpler matrices, which can also be regarded as decomposing the photon transport process into phases. One phase followed the photon pair from the annihilation point to the surfaces of the crystals, while a second phase accounted for all phenomena happening in the detector, including ICS. This latter phase used a detector transport probability map generated using Monte Carlo simulation.

Recently, deep learning solutions have become widespread in PET imaging, including ICS correction. Michaud et al. [8] were the first to show the use of neural networks to recover ICS events by analyzing triple coincidence data, achieving a 75% LOR recovery rate. They used a standard feedforward architecture with 4 layers (6, 10, 10, 5 neurons, respectively) and the $\mathop {tanh}$ activation function. Lee et al. [9] further advanced ICS recovery using CNN-based models, ICS-eNet and ICS-cNet, to estimate energy and interaction positions in pixelated PET detectors. Their solution showed positioning accuracy of up to 90% and substantial improvements in reconstructed image resolution.

Current ICS correction methods are typically performed in the LOR domain, since that is where ICS manifests. However, in ordered-subset expectation-maximization (OS-EM) [10], only a subset of the LOR data is available in each iteration, making on-the-fly LOR domain corrections difficult. To address this limitation, we propose a novel approach to ICS correction, which is performed in the image domain. Specifically, we train a multilayer perceptron on simulated data to estimate a $11 \times 11 \times 11$ filter kernel that captures the image-space effects of ICS. This filter is convolved with the image at the beginning of each OS-EM forward projection step. In addition to having no LOR data requirements during the reconstruction, the method also enables data-driven modeling of complex scattering patterns.

This article is an extended and improved work on the poster presented at *2025 IEEE Nuclear Science Symposium, Medical Imaging Conference, and Room Temperature Semiconductor Detectors Symposium*, Yokohama, Japan, November 2025. Major improvements include skew normal fitting in the filter data, application of position-dependent kernels instead of one aggregate kernel, larger filter width to better capture spatial information, as well as improved neural network architecture and training pipeline.

## Methods

Our solution uses a multilayer perceptron to learn an image-space filter from simulated LOR-space data. In every OS-EM forward projection, the current PET image estimate is convolved with this filter, thereby incorporating the model of ICS into the reconstruction.

We propose two strategies for creating the image-space filter. In the first approach, the neural network directly learns a $11 \times 11 \times 11$ filter kernel, with the constraints that all values are non-negative and the kernel sums to one. This allows for flexibility in the modeling of ICS. In the second approach, however, the network predicts the parameters of a 3D rotated skew normal distribution, which is then used to generate the $11 \times 11 \times 11$ filter kernel. Like the first method, this kernel is also normalized and non-negative, but it imposes a stronger structural prior, encouraging the filter to resemble a point spread function (PSF) of the 3D PET system.

### Skew normal distribution

The skew normal distribution generalizes the normal distribution by incorporating skewness. The univariate skew normal distribution [11] is defined as

1 $$\begin{aligned} \text {SN}(x; \mu , \sigma , \alpha ) = \frac{2}{\sigma } \cdot \phi \left( \frac{x - \mu }{\sigma } \right) \cdot \Phi \left( \alpha \cdot \frac{x - \mu }{\sigma } \right) , \end{aligned}$$

where $\phi$ is the standard normal probability density function (PDF) and $\Phi$ is its cumulative distribution function (CDF)

2 $$\begin{aligned} \phi (x) = \frac{1}{\sqrt{2\pi }} \; e^{-\frac{x^2}{2}}, \quad \quad \Phi (x) = \int _{-\infty }^{x} \phi (t) \; \textrm{d}t = \frac{1}{2} \left( 1 + \textrm{erf} \left( \frac{x}{\sqrt{2}} \right) \right) , \end{aligned}$$

$\textrm{erf}$ is the error function

3 $$\begin{aligned} \mathrm {\textit{erf}} \, (x) = \frac{1}{\sqrt{\pi }} \; \int _{0}^{x} e^{-\frac{t^2}{2}} \textrm{d}t, \end{aligned}$$

$\mu$ is the location (mean), $\sigma$ is the scale (standard deviation), and $\alpha$ is the skewness parameter.

The 3D version is constructed as the product of independent skew normal functions along each axis:

4 $$\begin{aligned} f(\textbf{x}) = \prod _{d=1}^{3} \text {SN}(x_d; \mu _d, \sigma _d, \alpha _d), \end{aligned}$$

where $\textbf{x} = [x_1, x_2, x_3]^\top \in \mathbb {R}^3$ represents a point in 3D space, and $\mu _d$, $\sigma _d$, and $\alpha _d$ are the location, scale, and skewness parameters for each axis $d \in \{1,2,3\}$.

To create an image-space filter kernel for PET reconstruction, the skew normal distribution is evaluated on a discrete 3D grid $\mathcal {G} \subset \mathbb {Z}^3$, centered at the origin with dimensions $11 \times 11 \times 11$ (that is, all coordinates are in $[ -5; 5 ]$). Each point $\textbf{x} \in \mathcal {G}$ is passed through the skew normal distribution and then normalized to ensure the kernel integrates to 1 so that it does not change the activity sum of the PET image:

5 $$\begin{aligned} k(\textbf{x}) = \frac{f(\textbf{x})}{\sum _{\textbf{x} \in \mathcal {G}} f(\textbf{x})}. \end{aligned}$$

To allow different orientations of the skew normal distribution in 3D space, we apply a rotation matrix $\textbf{R} \in \mathbb {R}^{3 \times 3}$ to the grid points prior to evaluation:

6 $$\begin{aligned} \textbf{x}' = \textbf{R} \textbf{x}, \quad \textbf{x} \in \mathcal {G}, \end{aligned}$$

7 $$\begin{aligned} \textbf{R}(\theta ) = \begin{pmatrix} \cos {\theta } & \sin {\theta } & 0 \\ -\sin {\theta } & \cos {\theta } & 0 \\ 0 & 0 & 1 \end{pmatrix}, \end{aligned}$$

where $\theta$ is the rotation angle, which is a learned parameter. Due to the cylindrical symmetry of the scanner geometry, we rotate only in the *xy* plane.

The parameters learned by the neural network are the location $\mathbf \mu = [\mu _1, \mu _2, \mu _3]^\top$, the scale $\mathbf \sigma = [\sigma _1, \sigma _2, \sigma _3]^\top$, the skewness $\mathbf \alpha = [\alpha _1, \alpha _2, \alpha _3]^\top$, and the rotation angle $\theta$. These are a total of 10 parameters, predicted by 10 neurons in the output layer. After prediction, the $\mu _d$ parameters are constrained to be in the range $[ -5; 5 ]$, the $\sigma _d$ parameters in the range [0.01; 10], the $\alpha _d$ parameters in the range $[ -5; 5 ]$, and $\theta$ in the range $[ 0^{\circ }; 360^{\circ } ]$. These ranges were selected for numerical stability and meaningful spatial representation of the voxel intensity distribution within a $11 \times 11 \times 11$ patch. If any output parameter is outside its valid range, it is clamped to the closest valid value.

**Fig. 2:**
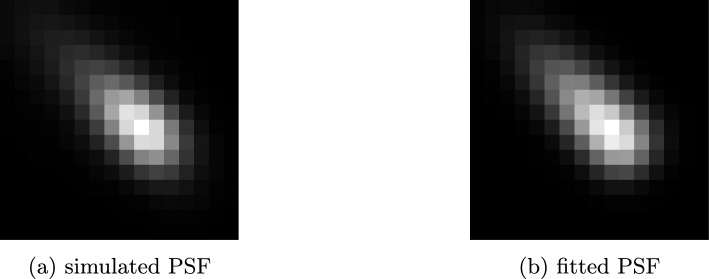
PSF generated by Monte Carlo simulation and PSF fitted with the rotated 3D skew normal distribution. Filter kernels learned for ICS correction are similar to PSFs, therefore skew normal distribution can be used for fitting

Figure 2a displays an approximate PSF for ICS on the nanoScan PET/CT (Mediso Ltd, Budapest) P122S scanner with 10 cm AFOV [12]. It was generated by forward projecting and backprojecting a point source placed in the origin, using Monte Carlo simulation of $10^8$ photon paths and detailed ICS modeling in the detectors. It can be observed that the PSF has an elongated, drop-like shape, which suggests that it can be effectively modeled using a rotated 3D skew normal distribution. Figure 2b shows a corresponding skew normal distribution, which had parameters of $\mathbf \mu = [3, 3, 0]^\top$, $\mathbf \sigma = [1.5, 5, 1.5]^\top$, $\mathbf \alpha = [0.5, -4, 0.5]^\top$, and $\theta = 316^{\circ }$. Note that the filter kernels learned for ICS correction are not necessarily the same as the direct physical PSFs. Instead, they represent effective correction operators embedded within the reconstruction pipeline. However, we use PSFs for illustration and approximate modeling due to their similarity.

### Neural network architecture and training

**Fig. 3:**
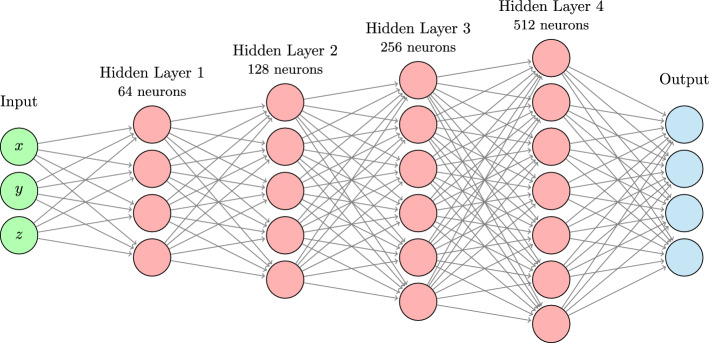
The architecture of the multilayer perceptron. In ICS-Net-direct, the output layer has $11 \times 11 \times 11 = 1331$ neurons, while in ICS-Net-skewnorm, the output layer has just 10 neurons used to parameterize the 3D skew normal distribution. In both cases, the final output is reshaped into a $11 \times 11 \times 11$ spatial filter kernel

We propose two neural networks for generating the image-space ICS correction filter:

1. *ICS-Net-direct*, which predicts the values of the $11 \times 11 \times 11$ filter kernel directly,
2. *ICS-Net-skewnorm*, which predicts the 10 parameters of the skew normal distribution presented in Sect. 3.1, which is then evaluated at grid points to obtain the $11 \times 11 \times 11$ filter kernel.

The two networks differ only in the output layers; the hidden layers, the input data, the training algorithm and every other aspect of the neural networks are the same. The kernel size of $11 \times 11 \times 11$ was selected via hyperparameter tuning, but very high reconstruction resolution may require a larger kernel size if the GPU memory allows it. Note that the skew normal distribution could be easily generalized by fitting parameters in world space, then sampling into the kernel size appropriate for the current voxel size and scanner resolution.

The input of both neural networks is a 3D spatial position in the measured volume, given in the physical coordinate system of the tomograph, where the origin is at the center of the tomograph and the unit of measurement is millimeters.

The training data were generated using Monte Carlo simulation. In each training sample, a position was sampled uniformly from the volume. A point source was placed in that position and the paths of $10^8$ gamma photon pairs were simulated, accounting for geometric forward projection and inter-crystal scattering. A data sample thus consisted of the 3D position and the LOR image forward projected with ICS.

Our Monte Carlo simulation tracked photon paths as follows. First, a random direction $\omega$ was sampled uniformly on the surface of a unit-radius globe. From the point source, two 511 keV photons were launched with directions $\omega$ and $-\omega$. If a photon hits the surface of a detector module, a free path length is selected randomly in accordance to the Beer–Lambert law. After the given path is traversed, a photon interaction takes place, which can be either photoelectric absorption, Compton scattering, or Rayleigh scattering. In the case of absorption, the photon path is terminated. In the case of Compton scattering, a new direction and energy is sampled from the Klein-Nishina distribution, and a new free path length is randomized. In the case of Rayleigh scattering, the new direction is sampled according to the Thomson cross section. The above process is repeated until termination, which occurs when the photon lost all of its energy or escaped the detector material. Photon hit is registered to the detector crystal that contains the interaction position in which the photon released the highest energy. This is typically the first interaction position in the detectors. If a photon goes through the detector modules without any interactions, it is discarded. Note that our Monte Carlo simulation does not include absorption or scattering in the measured volume, only in the detectors, since we have no information about the size, shape, or materials of the measured object. The neural network may learn more accurate filters if this information is available and modeled in the training data.

It should be noted that the simulation model assumes an idealized scanner geometry and detection environment. In practice, crystal imperfections and electronic noise introduce scanner-specific response patterns arising from small variations in materials and manufacturing processes. The proposed neural networks may be fine-tuned using measured data of a given scanner, resulting in a device-specific calibration step.

For training the networks, the voxel-space filter output needs to be forward projected into the LOR-space. Since the goal is for the filter to incorporate the effects of ICS, in this step we only perform a geometric forward projection, without ICS simulation. The resulting LOR image $y_\textrm{pred}$ is compared to the true LOR image $y_\textrm{true}$ (which simulated ICS in the LOR domain) and the smooth $L_1$ loss [13] is calculated as

8 $$\begin{aligned} \ell \left( y_\textrm{pred}, y_\textrm{true} \right) = [l_1, \dots , l_L, \dots , l_{N_\textrm{LORs}}]^T, \end{aligned}$$

9 $$\begin{aligned} l_L = {\left\{ \begin{array}{ll} \; 1/2 \; ( \, y_\textrm{pred}^{(L)} - y_\textrm{true}^{(L)} \,)^2 \; & \text {if } | \, y_\textrm{pred}^{(L)} - y_\textrm{true}^{(L)} \, | < 1 \\ \; | \, y_\textrm{pred}^{(L)} - y_\textrm{true}^{(L)} \, | - 1/2, & \text {otherwise} \end{array}\right. } \end{aligned}$$

An advantage of the smooth $L_1$ loss over the more commonly used $L_2$ loss is its reduced sensitivity to outliers, which can help mitigate the risk of exploding gradients. To further stabilize the training, we normalize the LOR images ($y_\textrm{pred}$ and $y_\textrm{true}$) by dividing each by its respective $L_1$ norm prior to loss computation.

The gradients for the backpropagation of the loss were approximated using geometric backprojection:

10 $$\begin{aligned} \nabla k \approx \textrm{backprojection} \left( \nabla \ell \left( y_\textrm{pred}, y_\textrm{true} \right) \right) , \end{aligned}$$

where $\nabla k$ is the gradient with respect to the filter kernel and $\nabla \ell$ is the gradient with respect to the smooth $L_1$ loss of the LOR image, which latter is calculated automatically by PyTorch [14]. Backprojection can be used to approximate gradients because it is the adjoint of the forward projection operator. (In our training pipeline, both forward and backprojection used only geometric projection without modeling further photon interactions, thus they can be considered to be a matched adjoint pair.) Since forward projection is expensive to differentiate, using the backprojection of the LOR-space loss to approximate the image-space gradient makes our solution more computationally efficient.

For the network architecture (Fig. 3), we used a multilayer perceptron with five fully connected layers: four hidden layers and one output layer. The hidden layers had 64, 128, 256, and 512 neurons, respectively, and were followed by Mish activation [15]:

11 $$\begin{aligned} a(x) = x \tanh \left( \ln (1 + e^x) \right) . \end{aligned}$$

In ICS-Net-direct, the output layer had $11 \times 11 \times 11 = 1331$ neurons and was followed by ReLU activation [16] defined as $a(x) = \max (0, x)$. ReLU ensures that the values of the filter kernel are non-negative. For ICS-Net-direct, the output neurons were initialized to predict a 3D isotropic Gaussian kernel ($\sigma = \frac{7}{6}$) in the first epoch.

In ICS-Net-skewnorm, the output layer had 10 neurons predicting the parameters of the rotated skew normal distribution. This is a regression task where both positive and negative values are possible, therefore no activation function was applied after the output layer. Instead, the skew normal distribution was evaluated on a $11 \times 11 \times 11$ grid to obtain the filter kernel (note that the values of the filter kernel can only be non-negative in this case as well). For ICS-Net-skewnorm, the output neurons were initialized to predict a 3D isotropic Gaussian-like kernel in the first epoch by using location $\mathbf \mu = [1, 1, 1]^\top$, scale $\mathbf \sigma = [1, 1, 1]^\top$, skewness $\mathbf \alpha = [1, 1, 1]^\top$, and rotation angle $\theta = 180^{\circ }$ starting parameters.

In both networks, the predicted filter kernel was normalized to ensure that its elements sum to 1. This normalization preserves the total activity in the PET image during convolution with the kernel in the OS-EM forward projection.

### Incorporating results into the OS-EM reconstruction

**Fig. 4:**
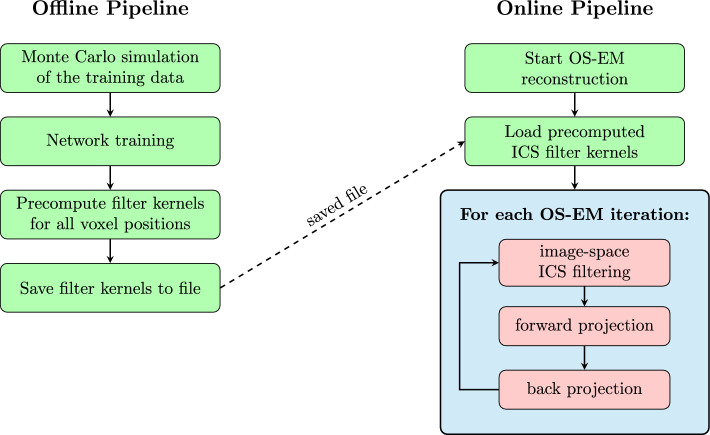
Method overview of the proposed image-space ICS correction pipeline. Position-dependent filter kernels are generated offline using a neural network trained on simulated data. During OS-EM reconstruction, the precomputed kernels are loaded and applied in an image-space filtering step before each forward projection

The trained neural networks produce a filter kernel for any 3D position in the measured volume. Before the OS-EM reconstruction, a filter kernel is precomputed and stored for each voxel center position. During reconstruction, these position-dependent filters are applied to the current PET image at the beginning of each forward projection step. This way, the correction of inter-crystal scattering is built into the reconstruction. An overview of our proposed pipeline is displayed by Fig. 4.

## Results

The proposed image-space ICS correction algorithm was evaluated on a Derenzo phantom [17] simulated using GATE [18] and on a rat acquisition measured on a nanoScan PET/CT (Mediso Ltd, Budapest) P122S scanner with 10 cm AFOV [12]. Reconstructions were using OS-EM with 8 subsets and 8 full iterations, and were performed on an NVIDIA GeForce RTX 4080 SUPER GPU. All algorithms were implemented using NVIDIA CUDA [19].

For the training data of the neural networks, 500 samples were simulated as described in Sect. 3.2. The data were used in 5-fold cross-validation, each fold placing 80% of the samples into the train set and 20% into the test set. The networks were trained for 50 epochs using the RAdam optimizer [20] with learning rate of $10^{-5}$ and batch size of 8.

The compared method were the following:

1. No ICS correction,
2. Image-space ICS correction using ICS-Net-direct,
3. Image-space ICS correction using ICS-Net-skewnorm,
4. LOR-space ICS correction (used as *ground truth*).

The LOR-space correction [21] consisted of high-sample Monte Carlo simulation of ICS in the LOR domain. It acts as a spatially invariant filter in the LOR domain, but its effects in the image domain are spatially variable, similar to our proposed method. It was chosen to be the ground truth because it has a long-standing use in PET reconstruction and has proven to achieve good image quality. Moreover, in real-world PET scans such as the rat acquisition, there is no better ground truth available.

For image quality measurement, we used the *normalized root mean square error*:

12 $$\begin{aligned} \textrm{NRMSE}(\textbf{x}_\textrm{pred}, \textbf{x}_\textrm{gt}) = \sqrt{\sum _{V = 0}^{N_\textrm{voxels}} \frac{( x_\textrm{pred}^{(V)} - x_\textrm{gt}^{(V)} )^2 }{ N_\textrm{voxels} }} \; \bigg / \; \langle \textbf{x}_\textrm{gt} \rangle , \end{aligned}$$

where $\textbf{x}_\textrm{pred}$ and $\textbf{x}_\textrm{gt}$ are the predicted and the ground truth voxel arrays, respectively, and $\langle \textbf{x}_\textrm{gt} \rangle$ is the average voxel intensity in the ground truth array.

### Derenzo simulation

**Fig. 5:**
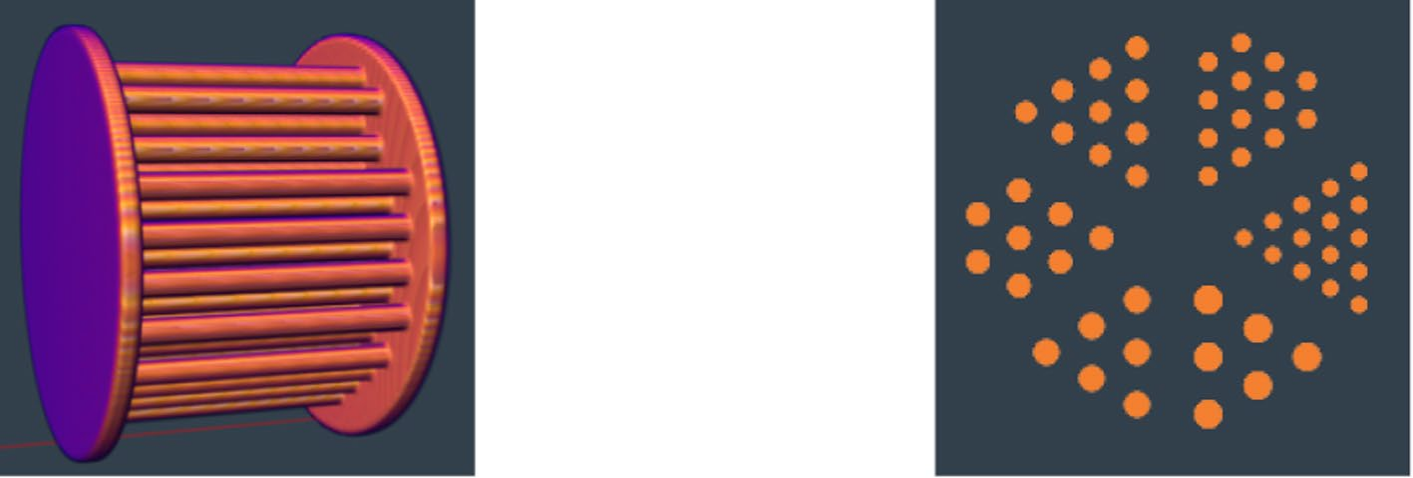
The Derenzo phantom has rods with varying diameters between two plates

**Fig. 6:**
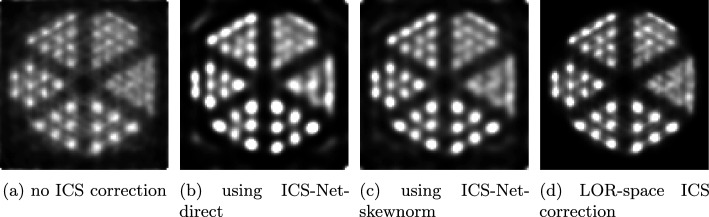
Reconstructed images of the Derenzo simulation with the different ICS correction strategies

The Derenzo phantom [17] (Fig. 5) is built of rods with varying diameters between two plates. The measured volume was divided into $100 \times 100 \times 100$ voxels. Figure 6 shows the reconstructed images for the different ICS correction methods.

Without ICS correction, the reconstructed image has low spatial resolution and low contrast due to the misplaced LOR endpoints. Applying image-space ICS correction visibly improves both metrics. However, ICS-Net-direct introduces a faint halo artifact around the phantom, while ICS-Net-skewnorm produces sharper edges and no artifacts.

The corresponding NRMSE values were:

- 0.87% using no ICS correction,
- 1.08% using ICS-Net-direct,
- 0.48% using ICS-Net-skewnorm.

On this phantom, ICS-Net-direct yielded a slightly higher NRMSE than without ICS correction, which is possibly due to the halo artifact. On the other hand, ICS-Net-skewnorm achieved a reconstructed image very close to that obtained with LOR-space ICS correction. This confirms that incorporating the skew normal distribution can improve the fidelity of the learned filters and may better capture ICS effects in some reconstruction targets.

### Rat acquisition

**Fig. 7:**
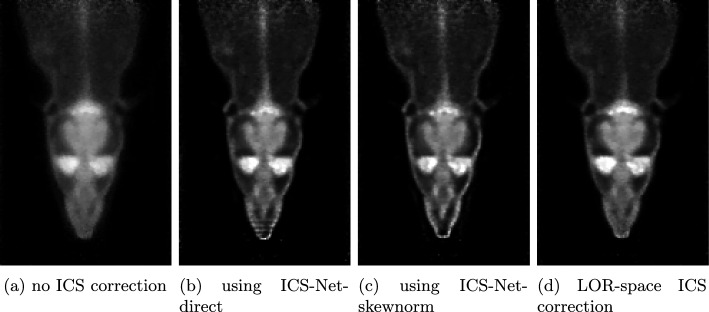
Reconstructed images of the rat acquisition with the different ICS correction strategies

In the rat acquisition, the measured volume was divided into $120 \times 90 \times 190$ voxels. Figure 7 displays the reconstructed images with the different ICS correction strategies.

Without ICS correction, anatomical details are hard to distinguish due to low spatial resolution. Both ICS-Net models greatly enhance the contrast of the reconstructed image and achieve even greater clarity of detail than using LOR-space ICS correction. However, ICS-Net-direct introduces mild staircase artifacts at the tip of the rat’s nose, while ICS-Net-skewnorm slightly underestimates the activity in the same region.

The NRMSE results were:

- 6.5% using no ICS correction,
- 2.92% using ICS-Net-direct,
- 4.08% using ICS-Net-skewnorm.

Both neural network approaches improve significantly over the uncorrected reconstruction. ICS-Net-direct achieves the lowest NRMSE, indicating that in complex reconstruction targets, such as living animals, the freedom of the filter kernel to model arbitrary LOR–volume correspondences is beneficial.

The runtimes of the full reconstructions were the following:

- 56.95s using no ICS correction,
- 60.98s using ICS-Net-direct,
- 60.71s using ICS-Net-skewnorm,
- 61.62s using LOR-space ICS correction.

Both ICS-Net variants run slightly faster than the LOR-space method, making image-domain ICS correction an efficient option for large-volume clinical data.

A comparison of all results is shown in Table 1.

**Table 1:** Comparison of ICS correction methods in terms of NRMSE and reconstruction runtime. LOR-space ICS correction was used as the reference method and therefore has no reported NRMSE value

Method	NRMSE (%)	NRMSE (%)	Runtime (s)
	Derenzo	Rat	Rat
No ICS correction	0.87	6.50	56.95
ICS-Net-direct	1.08	2.92	60.98
ICS-Net-skewnorm	0.48	4.08	60.71
LOR-space ICS correction	–	–	61.62

### Discussion

The presented results show that image-space ICS correction using learned filters is able to mitigate the resolution degradation caused by inter-crystal scattering. Even a simple neural network such as a multilayer perceptron can successfully learn to model complex photon interactions.

On the Derenzo phantom, ICS-Net-skewnorm achieved the lowest NRMSE (0.48%), outperforming both ICS-Net-direct and the uncorrected reconstruction. The resulting image displayed sharper rod edges as well as fewer artifacts and had an overall higher spatial fidelity. In contrast, ICS-Net-direct, although capable of capturing arbitrary kernel shapes, exhibited a halo artifact and a slightly higher NRMSE (1.08%) than the uncorrected case. This suggests that in simple, highly structured phantoms, an overparameterized kernel may overfit to noise or outliers in the training data. In this scenario, the skew normal kernel parameterization acts as a form of regularization.

In the rat dataset, both ICS-Net approaches significantly reduced the NRMSE compared to the uncorrected image, with ICS-Net-direct achieving the best score (2.92%). This result implies that the flexibility of direct kernels is advantageous in anatomically complex, real-world scans. ICS-Net-skewnorm, while still outperforming the uncorrected baseline (4.08% vs. 6.5%), slightly underestimated activity in certain regions. This could indicate a limitation of the skew normal representation when modeling highly nonlinear LOR–voxel mappings.

The image-space ICS filtering algorithm proved to be computationally efficient in a GPU-based setting. It introduced only a small increase in runtime (about 4s) compared to the uncorrected reconstruction and was slightly faster than the LOR-space correction method. This makes it well-suited for integration into time-sensitive clinical pipelines.

A key contribution of this work was the demonstration that effective image-space ICS correction can be achieved with minimal computational and implementation overhead, rather than surpassing a costly LOR-space method in absolute accuracy.

It may also be worth discussing why image-space ICS correction can be performed independently of other physical effects such as randoms, scattering in the object, or attenuation. First, random events are corrected in the LOR domain prior to any ICS correction, thus our method is not affected by it. ICS correction is also independent of attenuation, as only photons that ultimately reach the detectors can undergo inter-crystal scattering. A more relevant consideration is how changes in the photon energy spectrum caused by scattering within the object affect the resulting LOR-space inter-crystal scattering patterns. For example, if a photon’s energy is reduced to 400 keV after object scatter, the relative probabilities of Compton, Rayleigh, and photoelectric interactions in the detector change. However, in our previous experiments, we found that simulating ICS using photon energies in the 400–600 keV range, as opposed to assuming monoenergetic 511 keV photons, does not substantially alter the LOR-space scattering patterns or the final reconstruction results.

The limitation of our method is that it currently requires the precomputation of the filter kernels for every voxel position. This makes it necessary to generate a new set of filter kernels for each voxel resolution used in the reconstructions. (Note that this does not affect the neural network training, as that uses 3D world positions instead of discrete voxel indices.) Furthermore, the saved file size can be quite large for high voxel resolution. This could be avoided by integrating the neural network into the PET reconstruction framework, which we plan to implement in the future.

Another, intermediate solution could be to exploit the geometric symmetries of the PET scanner to substantially reduce the number of filter kernels that need to be stored. For typical scanner geometries, the detector arrangement and, consequently, the ICS-induced blurring patterns are symmetric with respect to the principal axes. Therefore, it is sufficient to compute and store filter kernels only for a single spatial octant of the image volume, while kernels for the remaining regions can be obtained by mirroring. This would reduce the required storage by approximately a factor of eight, lowering the memory size from 40 GB to 5 GB for a 200 × 200 × 200 volume, which is well within the capabilities of modern GPUs. In this solution, kernels corresponding to symmetry-related voxel positions could be averaged to obtain a more robust estimate of the ICS effect. Furthermore, assuming a smooth spatial variation of the learned kernels, an additional reduction in storage could be achieved by computing kernels on a coarser spatial grid and interpolating intermediate kernels as needed.

We also note that the network requires retraining when applied to another PET scanner model. In this case, the training data should be replaced with new data simulated using the new scanner geometry. Other modifications (e.g. modifying the training parameters) are not necessary.

## Conclusion

In this paper, we proposed an inter-crystal scattering technique for PET that operates entirely in the image domain. The approach uses a multilayer perceptron to predict spatial filter kernels which are then applied during the forward projection step of the OS-EM reconstruction. This process does not require access to LOR data during reconstruction, making it inherently compatible with iterative subset-based methods like OS-EM.

Two filter parameterization strategies were investigated: a direct 3D kernel representation (ICS-Net-direct), and a skew normal function fit (ICS-Net-skewnorm). Both approaches significantly improved image quality over the uncorrected reconstruction. ICS-Net-skewnorm performed best on the Derenzo phantom, while ICS-Net-direct on the rat scan. This suggests that the model should be selected based on the target application, that is, whether a structural, symmetric object or a complex, real-world object is to be reconstructed.

In the future, we plan to integrate the neural network into the Monte Carlo sampling procedures of the OS-EM forward projection. This would eliminate the need to precompute and store filter kernels for the voxel positions, making our solution faster, more accurate and more flexible. We also plan to investigate the generalization capability of the proposed solution into different types of scanner geometries.

When generating the training data for the neural network, we currently do not simulate absorption or scattering in the measured volume due to the lack of information about the target object. However, if Compton scattering takes place, the energy spectrum of the photons reaching the detectors changes. We plan to examine how approximating the target object with a cylinder of water improves the accuracy of the neural network’s predictions.

## Data Availability

The data that support the findings of this study are available from Mediso Medical Imaging Systems, but restrictions apply to the availability of these data, which were used under license for the current study, and so are not publicly available. Data are however available from the authors upon reasonable request and with permission of Mediso Medical Imaging Systems.

## Abbreviations

AFOV: Axial field of view
CNN: Convolutional neural network
CT: Computed tomography
GPU: Graphical processing unit
ICS: Inter-crystal scattering
LOR: Line of response
NRMSE: Normalized root mean square error
ML: Maximum likelihood
ML-EM: Maximum-likelihood expectation-maximization
OS-EM: Ordered subset expectation maximization
PDF: Probability density function
PET: Positron emission tomography
PSF: Point spread function
